# c-Myc and FOXO3a—The Everlasting Decision Between Neural Regeneration and Degeneration

**DOI:** 10.3390/ijms252312621

**Published:** 2024-11-24

**Authors:** Andrey M. Khaitin, Valeria V. Guzenko, Stanislav S. Bachurin, Svetlana V. Demyanenko

**Affiliations:** Laboratory of Molecular Neuroscience, Academy of Biology and Biotechnology, Southern Federal University, 194/1 Stachky Ave., Rostov-on-Don 344090, Russia; amhaitin@sfedu.ru (A.M.K.); vguzenko@sfedu.ru (V.V.G.); sbachurin@sfedu.ru (S.S.B.)

**Keywords:** c-Myc, FOXO3a, neurotrauma, neurodegenerative disorders, proteomics, intrinsically disordered proteins, post-translational modifications

## Abstract

The transcription factors c-Myc and FoxO3a play significant roles in neurodegenerative processes, yet their interaction in neurological disorders remains largely unexplored. In contrast, much of the available information about their relationship comes from cancer research. While it is well-established that FoxO3a inhibits c-Myc activity, this interaction represents only a basic understanding of a far more complex dynamic, which includes exceptions under specific conditions and the involvement of additional regulatory factors. Given the critical need to address this gap for the treatment and prevention of neurodegenerative disorders, this review consolidates current knowledge on the joint roles of these two factors in neuropathology. It also highlights their conformational flexibility, post-translational modifications, and outlines potential directions for future research.

## 1. Introduction

The balance between neural regeneration and degeneration is a critical determinant of neural health and function, with profound implications for recovery from neurodegenerative diseases and injury. Among the molecular pathways governing this balance, the interplay between the oncogene c-Myc and the forkhead transcription factor FOXO3a has emerged to be significant in determining cell fate [1].

Among the FOXO family proteins, FOXO3a appears to be the most important in neurodegenerative disorders due to its critical roles in regulating neuronal survival, apoptosis, and autophagy. It can activate pro-apoptotic pathways, leading to neuronal death, under conditions of severe stress or damage in neurodegenerative diseases [2,3]. FOXO3a enhances autophagy, a process that clears damaged proteins and organelles from neurons. This is particularly important in diseases like Alzheimer’s, where FOXO3a activation promotes autophagic clearance of toxic amyloid-beta (Aβ) aggregates [3,4]. FOXO3a helps to maintain cellular redox balance by regulating antioxidant defense genes. This is important since oxidative stress is a common feature of neurodegenerative diseases [2,3]. FOXO3a antagonizes the oncogene c-Myc, which promotes cell proliferation [2].

The transcription factor c-Myc, as well as transcription factors Oct4, Sox2, Klf4, is able to reprogram any somatic cell into induced pluripotent cells, as shown by the Nobel Prize Laureate Shinya Yamanaka [5]. c-Myc plays an essential role in cell cycle regulation, driving cell proliferation and growth by modulating genes involved in metabolism, protein synthesis, and mitochondrial function [6]. c-Myc regulates 10–15% of all genes in non-oncotransformed cells, influencing processes such as energy metabolism, protein synthesis, and the cell cycle [7]. In the nervous system, c-Myc is indispensable for neurogenesis, synaptic plasticity, and axonal growth [8]. Yet, its overactivation can lead to detrimental outcomes, including uncontrolled proliferation, impaired differentiation, and neuronal death [9,10]. There is mounting evidence that both acute and chronic injury to the central and peripheral nervous systems are associated with increased c-Myc activity [1]. c-Myc has a role in the early dysregulation of the neuronal cell cycle, which comes before neuronal apoptosis and degeneration [1,11], even though it is essential and sufficient for neural regeneration after axonal injury [12,13].

Some authors do, in fact., include c-Myc and FOXO3 in the same “axis” [14,15]. Both factors are well-studied in cancer biology, where they often oppose each other’s roles in proliferation and apoptosis. At the same time, their contributions to neural homeostasis and plasticity extend far beyond oncogenesis [10,16]. However, studies concerning both proteins and their interactions in the nervous system are almost absent. For example, searching the PubMed research query “(myc OR c-myc) AND foxo3* AND neur*” without any filters and specified fields yields only 10 results (dated 19.09.2024), where just about six are actually related to neurobiology. Since we cannot hope than this number would substantially increase to the date when this paper is published, it can be claimed that there is virtually no data on how the “axis” is specifically involved in neuropathological processes, and even if it is involved at all.

This article aims to provide an overview of the roles of these two transcription factors in neurological disorders related to neural regeneration and degeneration, their possible interactions, and the main prospects for future studies.

## 2. The Role of c-Myc and FOXO3a in the Regulation of Neuronal Regeneration in the Central and Peripheral Nervous System

**Figure 1:**
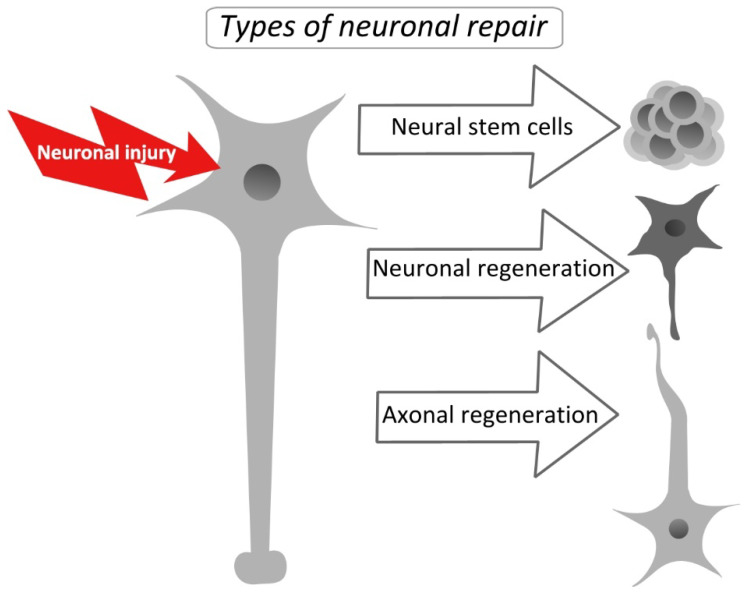
The three main types of neuronal repair after injury: axonal regeneration, neuronal regeneration, and stem cell-involving repair. See the details in the above text.

To date, it is believed that mammalian central nervous system (CNS) neurons have largely lost their regenerative capacity due to the need to preserve their synaptic structure to protect the brain’s cognitive processes [17,18]. The cranium’s durability on the outside and additional buffering by glial cells provide the brain with sufficient mechanical protection. This allowed mammals to develop high cognitive abilities in the course of evolution, which was probably prioritized over preserving the regenerative potential of the CNS. However, in neurodegenerative diseases and various CNS injuries, like strokes or brain and spinal cord injury, even imperfect strategies for axon regeneration and restoration of synaptic contacts can be immensely beneficial. In the adult mammalian CNS, axotomy causes neuronal degeneration and/or death. Due to the reduced regenerative capacity of adult neurons and the presence of inhibitory factors in the environment, spontaneous axonal regeneration rarely occurs [19]. Comparative studies on the causes of the differences between the ability to regenerate axons in young and adult individuals have identified some regulators of regeneration, such as, for example, cAMP, whose levels are significantly reduced in mature neurons [20]. Numerous findings, however, suggest that many intact neurons have a considerable capacity for structural plasticity and axon sprouting, even in the mature central nervous system [21]. They even have the ability to return to a development state similar to that of embryos, which permits axon regeneration [22]. There are three different ways that damaged nerve tissue might be repaired (Figure 1). The regeneration of injured sensory or motor nerve axons from the intact neuronal body is the most easily achieved kind of recovery [23,24]. Both the creation of synapses and the activation and regulation of axon development are necessary for this process. Because it includes repairing the remaining injured neuron, the second form of restoration is more challenging to complete [25,26]. A certain degree of neuronal functional integrity is necessary. The third and least likely mode of regeneration necessitates the presence of multipotent neural stem cells in order for new neurons to develop to replace damaged neurons [27,28] (Figure 1). Moreover, the surrounding tissue must retain the ability to ensure migration, growth, and synapse formation, which is necessary for the restoration of neuronal networks. Most of the time, the surviving neurons regenerate but no new cells are formed [1].

c-Myc can play both pathologic and physiologic roles in nerve cell injury [9,10]. Yet, there is a clear dichotomy between the PNS and CNS regarding the reparative action of c-Myc. What might be the mechanisms responsible for the same type of physical injury causing an increase in c-Myc levels in PNS neurons, but a decrease in c-Myc expression in CNS neurons [1]? One reason for these differences may be the differences in neuronal responses to acute stress, which depends on a strict relationship between the levels of FOXO (forkhead box) and MYC transcription factors. FOXO proteins repress MYC activity and compete with it for binding promoter regions of genes involved in cell cycle progression [29,30].

**Figure 2:**
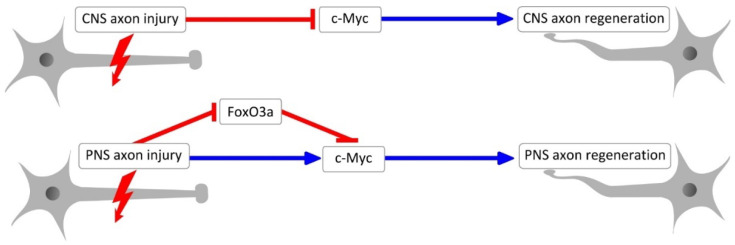
Schematic involvement of c-Myc and FOXO3a transcription factors in axon injury response and regeneration. Blue sharp-headed arrows indicate promotion, red blunt-headed arrows indicate inhibition.

Levels of FOXO3a (forkhead box O3a) and its downstream target p27 (kip1) rise somewhat after injury, but subsequently sharply decline, according to data from a sciatic nerve compression model [16,31]. In many neuronal subtypes, the activation of apoptotic genes was first associated with the stress-induced nuclear translocation of the transcription factor FOXO3a. It has been suggested that FOXO3a has a more intricate function in how sensory neurons react to damage. FOXO3a expression and its nuclear localization were evaluated in adult rat DRG neurons ipsilaterally, contralaterally, or distant from the site of damage in a model of chronic unilateral transection of the L4-6 spinal nerve in comparison to intact animals [16]. Both nuclear and cytoplasmic FOXO3a levels are elevated in healthy neurons. Furthermore, a peptide associated with the calcitonin gene, a hallmark of the nociceptive subpopulation of neurons, colocalizes with the protein. Nuclear FOXO3a in damaged tiny neurons increased dramatically within an hour of injury, then significantly decreased within 1, 2, and 4 days. One week after axotomy, the levels were no different from those in undamaged mice. In intact contralateral and remote-from-injury neurons, a stronger biphasic response to injury was seen. The damaged neuron was shielded from apoptosis by the early reduction in FOXO3a expression and nuclear localization [16]. Thus, decreased FOXO3a levels in sciatic nerve injury may promote c-Myc activation and axon regeneration during a time period, while translocation of FOXO3a to neuronal nuclei may prevent the realization of the proliferative potential of c-Myc during another one. According to several researchers, the intricate relationship between FOXO3a and c-Myc during axon regeneration is shown in the surrounding glial cells, distant neurons, and injured neuronal cells. It is also likely not a quick process [1]. Glial cells undergo apoptosis within the first few days of peripheral nerve injury, although neuronal apoptosis does not occur in the short term. Only one month following axotomy can the retrograde loss of L4-5 DRG sensory neurons be identified, because glial cells provide autocrine and trophic support to the injured nerve [32,33]. At the same time, unfortunately, there are currently no data describing the dynamics of FOXO protein changes in CNS axon injury. Taking into account the role of FOXO and MYC proteins in axon regeneration [34] and their competitive relationships, it would be reasonable to compare the dynamics of expression and intracellular distribution of FOXO and MYC in CNS neurons upon injury. See Figure 2 for a summary of the mentioned facts.

In the central nervous system, FOXO3 also has a critical role in maintaining the quiescent state of neural stem cells in the adult mouse brain. The deletion of Foxo3 causes early neural stem cell differentiation, which reduces the neural stem cell pool and causes abnormalities in brain development [35].

## 3. Involvement of c-Myc and FOXO3a in the Development of Neurodegenerative Diseases

A key feature of neurodegenerative diseases, particularly Alzheimer’s disease (AD), is cell death, while cancer, in contrast, involves uncontrolled cell proliferation, which puts these two pathologies on opposite sides of the spectrum of cellular population dynamics. What is interesting, however, is that both processes begin with the induction of cell proliferation. Trauma, stress, or viruses cause cell cycle activation in neurons just as they do in other cell types. However, unlike cell types with significant proliferative potential, where disruption of cell cycle regulation can cause malignant transformation, in neurons, cell cycle activation most often leads to their death and less often to the induction of DNA replication and tetraploidy of a fraction of neurons, which is an early sign of neurodegeneration in the adult brain [36].

So, in AD, damaged neurons become susceptible to degeneration when attempting to re-enter the cell cycle [37]. Along with amyloid plaque development, tau hyperphosphorylation, and apoptotic neuronal death, the poorly controlled attempt by neurons to re-enter the cell cycle is currently regarded as a characteristic of AD [38,39,40]. Other neurological disorders that accompany neuronal degeneration also exhibit alterations in the neuronal cell cycle. Therefore, it is possible that neurons re-enter the G1 phase of the cell cycle in Parkinson’s disease (PD) and amyotrophic lateral sclerosis due to the accumulation of hyperphosphorylated pRb (retinoblastoma protein) in neurons and changes in the location of the E2F1 transcription factor [41,42]. According to the current cell cycle hypothesis, diseases with insufficient cell cycle control include cancer’s cell proliferation and AD’s neurodegeneration [39]. Curiously, a large longitudinal study with over a million participants discovered that Alzheimer’s disease and cancer do not usually co-occur, particularly in older adults. When AD is present, the chance of acquiring cancer can be lowered by up to 50%, and in cancer patients, the risk of developing AD can be lowered by up to 35% [43]. A recent study [44] connects the pathogenetic cell-cycle reentry with aging. Using a bioinformatics strategy, it was revealed that neurons which had undergone such an event become senescent and could accumulate in neurodegenerative disorders and contribute to their development.

Evidence suggests that c-Myc, a key player in determining cell destiny, contributes to the early disturbance of neuronal cell cycle control that occurs prior to dementia. Dystrophic neurons and neurites with neurofibrillary tubules have been found to contain phosphorylated active c-Myc protein in a number of neurodegenerative tauopathies, such as AD, Pick’s disease, progressive supranuclear palsy, and cortico-basal degeneration, which is linked to neuronal damage and apoptosis [45,46]. This raises the question of whether absence of co-occurrence between AD and cancer be due to variations in MYC activity. According to certain theories, the degree and duration of energy stress play a crucial role in the onset of both AD and cancer, even if cellular stress may serve as a common trigger for both conditions [47]. High-energy-dependent neurons are primarily affected by the extreme acute energy stress that arises during the development of cancer. This causes mTOR to be activated and additional ROS to be produced, which in turn activates the FOXO family of transcription factors, primarily FOXO3a, increasing the levels of antioxidant defense enzymes to prevent possible neuronal damage [48,49]. FOXO1 and FOXO3a proteins suppress c-Myc activity [29,30]. However, under chronic stress, the protective role of FOXO is ignored and c-Myc activity is not suppressed, leading to neurodegeneration and the development of AD [47]. The repressor protein MM-1 (MYCModulator-1), which regulates c-Myc activity in addition to FOXO-related pathways, also influences neurodegenerative processes through mechanisms of protein quality regulation and aggregation control, including amyloid aggregation [11]. Additionally, the NMDA receptor-dependent apoptotic cascade, which culminates in NF-kB and p53 activation, is downstream of c-Myc [50,51].

How can the various roles of c-Myc in neuronal regeneration and degeneration be explained? Recent research has shown that the cytoplasmic form of the protein has a hitherto underappreciated role in enhancing cell survival, even though c-Myc activity has primarily been ascribed to its function as a transcription factor.

**Figure 3:**
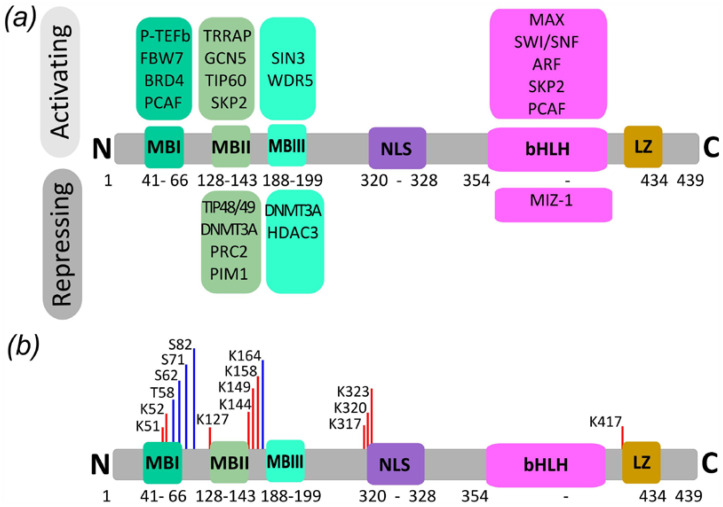
Domains and PTM sites of c-Myc. (**a**) Domain structure and interaction partners of c-Myc (N-terminal transactivation domain TAD including MBI and MBII partner interaction domains, nuclear localization sequence (NLS) and an intrinsically disordered C-terminal region containing the bHLHZip domain together with the binding sites of its key partners MAX, MIZ1, ARF, and SKP. (**b**) Known phosphorylation and acetylation sites of c-Myc according to UniProt data (http://www.uniprot.org/ (accessed on 20 September 2024)).

C-Myc consists of 439 amino acid residues and has several conserved regions that are the same in all members of the Myc family. It consists of an N-terminal transactivation domain (TAD) including MBI, MBII and MBIII partner interaction domains, nuclear localization sequences (NLS) and an intrinsically disordered C-terminal region containing the bHLHZip domain together with the binding sites of its key interaction partners (MAX, MIZ1, ARF, SKP) and DNA-binding domains. These domains contain highly conserved modules that provide docking sites for a large number of cofactors that regulate the activity and stability of c-Myc (Figure 3).

As studies have shown [52,53], the cytoplasmic form of c-Myc, Myc-nick, can be formed by calpain-dependent proteolysis of full-length c-Myc at lysine 298. Myc-nick retains the conservative Myc box regions but lacks the nuclear localization signal (NLS) and the bHLHZ domain required for binding to Max and DNA. Myc-nick is required for cell survival during muscle differentiation. The inhibition of c-Myc proteolysis prevents differentiation of myoblasts into myotubules and induces apoptosis [52]. c-Myc is constitutively converted to Myc-nick in the cytoplasm of most normal and cancer cells. However, in cells exposed to environmental stress, which includes high cell density, hypoxia, and nutrient deprivation, Myc-nick is the predominant form of c-Myc [53]. The N-terminal region of c-Myc is a binding site for acetyltransferase recruitment and for binding to α-tubulin [54]. Myc-nick binds to α-tubulin and induces acetylation of α-tubulin by recruiting the histone acetyltransferase GCN5 to microtubules. It has been shown that during muscle cell differentiation, levels of Myc-nick and acetylated α-tubulin increase, while levels of full-length Myc decrease, leading to cell reorganization and differentiation [52]. Acetylation of α-tubulin at lysine 40 stabilizes microtubules and promotes their flexibility [55]. In neurons, acetylated α-tubulin is involved in both dendrite morphogenesis and axon growth. In murine models, the ablation of MEC-17 (α-TAT1), which leads to the loss of α-tubulin acetylation, has been shown to induce excessive axon branching and outgrowth [56]. The ability of the N-terminal segment of c-MYC to bind to acetyltransferases appears to be critical for the biological functions of c-Myc in the cytoplasm. Deletion of the MBII region (amino acids 106-143) required for binding of c-Myc to acetyltransferases GCN5 and TIP60 reduces the ability of c-Myc to promote cancer cell survival, chemoresistance, and migration [53]. Thus, the nuclear and cytoplasmic forms of c-Myc may have directly opposite effects on survival and regulation of neuronal regeneration after injury and during degeneration. Moreover, while quite a lot is known about c-Myc as a transcription factor, almost nothing is known about the Myc-nick protein cytoplasmic form and its role in neuronal regeneration and degeneration.

It is unknown how c-Myc expression and activity in the PNS and CNS are controlled during regeneration or degeneration, but one mechanism is likely to be the interconnection between FOXO1/3 and c-Myc protein levels [57,58].

Similar to c-Myc, the activity of its antipode, FOXO3a, also depends largely on the intracellular localization of the protein. FOXO3a is predominantly regulated through its phosphorylation by Akt kinase. The activation of the PI3K/Akt pathway is triggered by many stimuli, including the action of insulin, insulin-like growth factor, epidermal growth factor, and erythropoietin. Phosphorylation of FOXO3a by T32 and S253 leads to the formation of binding sites for 14-3-3 proteins that promote the exclusion of FOXO3a from nuclei, thereby blocking its role as a transcription factor and promoting its translocation to the cytoplasm. Phosphorylation of FOXO3a by casein kinase 1 (CK1) at S315 results in accelerated cytoplasmic sequestration of the protein [59]. Tyrosine-phosphorylated regulated dual-specificity regulated kinase 1A (DYRK1A) has been shown to phosphorylate FOXO3a, which also promotes its cytoplasmic translocation [60]. The phosphorylation of FOXO3a is reversible; the PP2A protein phosphatase dephosphorylates FOXO3a, allowing its reentry into cell nuclei. Many anticancer drugs (e.g., cisplatin) induce dephosphorylation and acetylation of FOXO3a [61]. The reduction of endogenous FOXO3a levels by RNA interference renders hippocampal neurons more resistant to excitotoxicity. A number of stimuli, including stimulation of extrasynaptic N-methyl-D-aspartate receptors, reduced growth factors, and oxygen–glucose deprivation caused rapid translocation of FoxO3a from the cytosol to the cell nucleus, inducing neuronal cell death. This translocation was inhibited in hippocampal neurons that were subjected to prolonged periods of synaptic activity prior to injury and was dependent on the activity of calcium/calmodulin-dependent protein kinase IV [62]. The inhibition of JNK (c-Jun N-terminal kinases) activity can inhibit the translocation of FOXO3a from the cytoplasm to the nucleus and reduce the levels of pro-apoptotic proteins Bim and CC3, resulting in reduced neuronal apoptosis after hypoxic-ischemic brain injury [63]. Also, like in the case of c-Myc, the functions and physiological effects of the nuclear and cytoplasmic forms of FOXO3a are very different.

In AD, FOXO3a is significantly upregulated in the brain, promoting Aβ production and neurodegeneration. However, it also plays a protective role by upregulating stress-resistance genes, thereby defending neurons from Aβ-induced toxicity [2,4]. Additionally, c-Myc is implicated in cell cycle processes that contribute to AD progression [1]. In Parkinson’s disease (PD), FOXO3a is linked to Lewy bodies and contributes to dopaminergic neuron death, with its activity involved in ROS detoxification during oxidative stress [2,64]. In Huntington’s disease (HD), FOXO3a is overexpressed and appears to regulate its own expression through a positive autoregulation loop, potentially initiating a protective response to cellular stress [64]. By suppressing c-Myc, FOXO3a helps reduce reactive oxygen species (ROS) levels, which is vital for neuronal health, as elevated ROS can cause oxidative damage and apoptosis [65].

**Figure 4:**
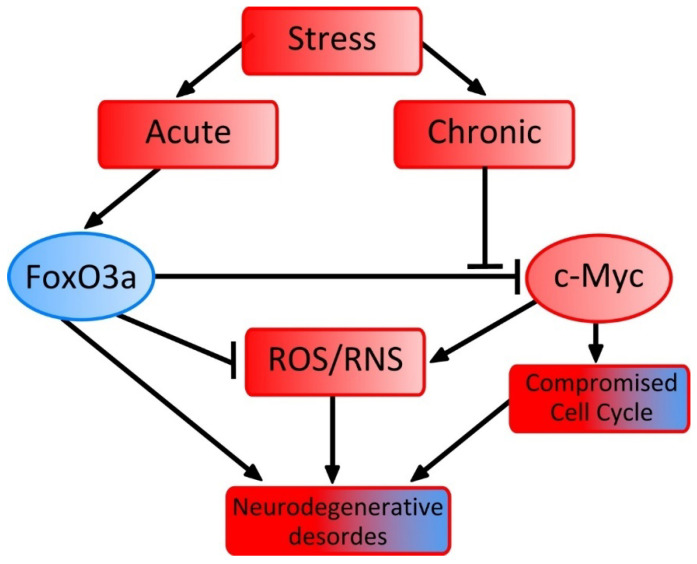
Schematic interactions of c-Myc and FOXO3a in the pathogenesis of neurodegenerative disorders. Damaging factors are indicated in red. Transition from blue to red indicates dysregulation of cellular functions. Sharp-headed arrows indicate promotion, blunt-headed arrows indicate inhibition.

The relations of the two transcription factors are summarized in Figure 4. In the boxes, red indicates damaging factors, and blue-red gradients indicate dysregulation of cellular functions. However, it should be noted that although FOXO3a inhibits c-Myc under certain conditions, it can also promote c-Myc transcription in specific contexts. For example, FOXO3a has been found to activate c-Myc expression by directly binding to its promoter region, indicating a complex regulatory role that may vary depending on the cellular environment [66]. Myc is also involved in naive and memory T cell activation in multiple sclerosis [10]. Concurrently, an expanding evidence suggests that Foxo3a plays a role in the pathogenesis of multiple sclerosis (MS) through pro-apoptotic mechanisms, as well [67,68].

## 4. Intrinsically Disordered Proteins of the c-Myc and FOXO Families

The 20th century saw the establishment of the paradigm for protein structure and function. This paradigm’s main idea is that a protein’s function depends on its ordered (rigid) and distinct three-dimensional structure [69]. A protein’s amino acid sequence encodes its three-dimensional native structure, which is at the core of the structure–function paradigm. This structure is controlled by a complex balance of different physical forces between the protein’s atoms. This paradigm’s acceptability has been aided by the growth of the enzyme catalysis hypothesis and knowledge of how receptors, transporters, membrane channels, etc., function [69]. Over the years, our understanding of the structure and function of proteins has greatly expanded. It turns out that approximately 30% of all mammalian proteins are disordered, i.e., lack stable secondary and tertiary structure disordered, whereas 75% of all signaling proteins contain extended disordered regions. Such proteins with unstable significant regions (or along their entire length), have been termed intrinsically disordered proteins (IDPs) [69]. In order to carry out their signaling tasks, IDPs momentarily attach to a number of interaction partners in dynamic regulatory networks that can digest complicated information and react accurately and quantitatively to cellular signals. Temporal and dynamic molecular interactions occur when IDPs trade binding partners and compete for binding with central hub proteins, which are frequently found in small amounts and have brief half-lives. These proteins can behave as switches and rheostats because post-translational changes fine-tune these interactions [69,70,71].

Multifunctional proteins that lie at the center of a number of signaling pathways and act as central hubs in cell signaling networks usually contain intrinsically disordered regions (IDRs) that facilitate the dynamic assembly of triple and higher-order complexes and integrate diverse signaling pathways [72,73]. C-Myc, as well as its antagonist FOXO3a, stands at the interface point of several intracellular signaling cascades and belongs to a group of multifunctional proteins with a disordered structure [6,74]. Both c-Myc and FOXO play key roles in cell fate determination, though with opposite effects [65]. Growth factors, metabolic stress, oxidative stress, and other cellular stimuli send signals to both proteins, which then translate those signals into responses that alter protein-protein interactions and spatiotemporal gene expression, either causing apoptosis or promoting repair and regeneration [73,75,76]. Reversible post-translational modifications (PTMs), like as phosphorylation, acetylation, and ubiquitination, precisely control these activities. Depending on the situation, these PTMs might interact and even have opposite effects [77].

The c-Myc N-terminal TAD domain is a disordered region of the protein that interacts with hundreds of proteins regulating chromatin rearrangement, transcription, stability, and interaction with partners [78]. In cell nuclei, c-Myc can acquire an ordered structure after binding to its disordered partner protein MAX. As predicted by the disorder prediction algorithm, c-Myc is expected to contain more than 45% of residues that are prone to forming a disordered structure. Nuclear magnetic resonance studies of the disordered region of c-Myc (residues 1-88, c-Myc boxes MBI and MBII) demonstrated its functional plasticity and capacity to form multiprotein complexes. The N-terminal domain encompasses residues 1 to 167 and is linked to the C-terminal region by a flexible linker. This region of TAD serves as the central hub for all transactivation activity, which is regulated by binding to multiple proteins. The significance of Myc-boxes in cellular transformation and proliferation has been substantiated by experiments employing targeted mutagenesis. Nevertheless, the precise biochemical mechanism through which c-Myc-mediated transactivation occurs remains elusive, largely due to the paucity of structural data pertaining to the TAD region. However, studies have identified the residues spanning 1 to 143 within the TAD region as indispensable for neoplastic transformation, differentiation, and apoptosis [79,80].

Like c-Myc, FOXO3a is particularly rich in IDRs lacking clearly determined 3D conformations (predicted to be up to 75% of the protein sequence) [81] Human FOXO3a is a 673 amino acids protein with an N-terminal FH domain (forkhead domain, 157-237 amino acid residues) that contains a winged helix fold, and the rest of FOXO3a is highly disordered, although these IDRs contain three regions (CR1-CR3) conservative among FOXO family proteins, which, include the TAD domain [82]. Thus, the overall conformation of FOXO3a resembles a rigid “head” followed by a flexible “tail”. The FOXO3a molecule exists in a “closed” conformation where both DBD and TAD are partially autoinhibited [83].

The disordered structure of c-Myc and FOXO family proteins indicates that both proteins may play a key role in determining cell fate with opposite effects. The presence of a disordered structure facilitates the dynamic assembly of protein-protein complexes of different orders involving both proteins, integrating diverse neuronal signaling pathways in response to external stimuli. It is likely that the complex competitive interactions between FoxO1/3a and c-Myc or its shortened form during neuronal regeneration or degeneration allows c-Myc to realize its regenerative potential while limiting its proliferative potential. An additional level of functional and structural regulation of proteins with disordered structure, to which c-Myc and its antipode FoxO1/3a belong, is provided by posttranslational modifications.

## 5. Post-Translational Modifications of c-Myc and FOXO Family Proteins

The acetylome of cells, especially those not oncotransformed, is much less studied than the phosphoproteome [84]. At the same time, acetylation sites are highly conserved, much more conservative and evolutionarily older than phosphorylation sites. Furthermore, a comparative analysis of lysine conservation in Drosophila and humans with respect to that in nematodes and Danio rerio fish revealed that acetylated lysins exhibited significantly greater conservation than non-acetylated lysins. Bioinformatic investigation employing gene ontology terms indicated that proteins bearing acetylation-conserved residues regulate essential cellular processes, including translation and folding of proteins, DNA packaging, and mitochondrial metabolism [85]. At present, the number of post-translational modifications (PTMs) identified through mass-spectrometric techniques greatly exceeds the number of PTMs for which functional understanding exists. The situation is similarly underdeveloped in the case of phosphorylation, where functional information exists for fewer than 3% of mapped phosphorylation sites in the human proteome. This figure is considerably lower for acetylation sites. In our view, a significant challenge for modern proteomics in the post-genomic era is to gain insight into the poorly understood phosphorylation, as well as other PTMs such as acetylation. A number of complementary approaches may be employed to this end, including the use of existing multi-omics data to predict functionality or the screening of mutant libraries under different physiological conditions. Alternatively, the use of functional proteomics to assess the impact of PTMs on protein properties represents a promising, more focused approach. With the advent of machine learning algorithms, it is now possible to employ an in silico approach to predict PTM sites and the potential biological effect [86]. And the combination of predicting sites of a particular modification with the capabilities of molecular dynamic simulation of protein conformation and docking, allows for a more directed and meaningful approach to planning in vitro and in vivo experiments. Our studies using this approach show, for example, that point acetylation of p53 at lysine 320 by PCAF acetyltransferase can promote p53 translocation between the nucleus and cytoplasm in penumbra neurons with preferential accumulation in the neuronal cytoplasm. Additionally, p53 acetylation at lysine 320 is more preferential than acetylation at lysine 373 and supports penumbra neuron survival and recovery following photothrombotic stroke. Therefore, methods to increase p53 acetylation at lysine 320, either by inhibiting HDAC1 or HDAC6 deacetylases or by increasing PCAF activity, or a combination of these measures, may be therapeutically beneficial for post-stroke recovery [87].

Recent studies indicate that c-Myc can be acetylated by a number of cAMP response element-binding protein (CREB) acetyltransferases [88]. Its acetylation by p300, GCN5, and TIP60 partially stabilizes c-Myc via suppression of ubiquitin-dependent degradation of the protein and promotes its interaction with partners such as MAX [89]. On the other hand, hyperacetylation of c-Myc induced by administration of HDAC inhibitors destabilizes the protein [90], and deacetylation of c-Myc at lysine 323 by HDAC3 in cholangiocarcinoma cells protects c-Myc from ubiquitin-dependent proteolysis [91]. Regrettably, most studies on the effect of post-translational acetylation of c-Myc on its activity have been performed on oncotransformed cells; little is known about the effect of acetylation on the properties of c-Myc in nerve cells. Our studies suggest that in photothrombotic penumbra neurons there is an increase in acetylation of c-Myc at the N-terminal lysine 148 but not at the C-terminal lysine 323. The results of molecular dynamic simulations suggest that the lysine at position 148 of c-Myc plays a key role in stabilizing the spatial structure of the protein. The ischemia-induced increase in the level of acetylation of c-Myc at lysine 148 by acetyltransferase p300 may prevent the protein from binding to its partner MAX in cell nuclei, preventing it from realizing its proapoptotic potential and promoting its cytoplasmic translocation. The acetylation of c-Myc at lysine 148 in the cytoplasm had no effect on its interaction with α-tubulin [92].

The combination of different PTMs is crucial for the function of FOXO family proteins. There is even a concept as a “FOXO code”, similar to the “histone code”, which combines several PTMs of proteins to regulate their functions [75,93]. The major PTMs of lysine residues are acetylation, methylation, and ubiquitination. However, the process of lysine ubiquitination is seldom directly targeted by protein regulation. Rather, the addition or removal of other modifications at or in close proximity to the ubiquitination site typically occurs prior to this event. These PTMs serve as recognition sites, thus providing an additional level of rapid and reversible regulation and fine-tuning prior to the irreversible degradation of the protein. The experimentally observed number of sites in the protein for various PTMs greatly exceeds the number of protein-coding genes in the human genome. For example, a recently conducted study of lung cancer using proteomics revealed that approximately 35% of phosphorylated proteins also exhibited a minimum of one or two methylation or acetylation sites. Consequently, individual PTMs of a particular protein are increasingly being regarded as integral components of regulatory networks, whereby combinations of PTMs act in concert to achieve specific functional outcomes [94]. According to the data, PTMs may act as exclusive XOR switches for diverse biological effects when there are inverse correlations between them. Phosphorylation versus acetylation, phosphorylation versus methylation, and methylation versus acetylation are all covered by this idea. Indeed, acetylation and methylation sites have also been shown to exhibit negative associations within proteins. By reversibly changing the sets of interacting proteins with their domains, acetylation, methylation, and phosphorylation enhance the interactome of hub proteins in signal transduction networks. The acetylation of FoxO1 at lysins 242 and 245, and FoxO3 at lysins 245 and 248 significantly reduces the proteins’ ability to bind to DNA and promotes cytoplasmic localization of FoxO proteins, potentially protecting neurons from damage. CBP/P300 acetyltransferase acetylates FoxO proteins in response to oxidative stress. On the other hand, the deacetylation of FoxO family proteins by histone deacetylases SIRT1, SIR2, SIRT3, or HDAC3 promotes the nuclear localization of the protein and contributes to its function as a transcription factor [57]. In general, it is believed that acetylation of FOXO3a promotes cell survival, whereas deacetylation by, for example, SIRT1 promotes autophagy [75].

The phosphorylation of FOXO3a at specific sites, like Thr32 and Ser253, enhances its binding to 14-3-3 proteins, promoting its retention in the cytoplasm and reducing its nuclear localization and transcriptional activity. When phosphorylated, FOXO3a is less capable of effectively interacting with c-Myc, as it is sequestered away from the nucleus where c-Myc operates [95,96]. FOXO3a activation has been shown to increase c-Myc phosphorylation at T58, a modification crucial for c-Myc recognition by the Fbw7 ubiquitin ligase, leading to its enhanced degradation. This suggests that FOXO3a indirectly regulates c-Myc levels through its phosphorylation state [97]. Additionally, acetylation of FOXO3a by coactivators like CBP/p300 can influence its transcriptional activity. Acetylated FOXO3a may exhibit altered DNA-binding abilities, modulating its interaction with c-Myc. In particular, acetylation near phosphorylation sites can alter the conformation of FOXO3a’s nuclear localization signal (NLS), affecting its ability to bind DNA and interact with transcription factors like c-Myc [95,98]. Furthermore, acetylation exposes lysine residues on FOXO3a, making them targets for ubiquitination. This modification can lead to increased degradation of FOXO3a or influence its interaction with c-Myc. For example, acetylation may make FOXO3a more susceptible to ubiquitin-mediated degradation, thereby reducing its availability to interact with c-Myc [96,98].

Thus, even pointwise PTM of multifunctional, intrinsically disordered proteins, such as p53 [87,99], MYC [92,100], FOXO [75,96], E2F1 [84,101], and a number of others, which are hubs of several signaling pathways in the cell, can have a significant effect on protein conformation, localization, and interaction with partners, which may ultimately lead to the implementation of diametrically opposite functions. More and more data confirm the important role of such hub proteins in the development of cardiovascular diseases, cancer, and neurodegeneration [75,102,103].

## 6. Future Prospects

Given the above findings, further investigation of the mechanisms of regenerative and antidegenerative activity of c-Myc is needed. Is the detrimental c-Myc activity in neurodegeneration simply a failed attempt to protect and repair the CNS from damage? How can the regenerative and antidegenerative potential of the Yamanaka factor protein be effectively managed? By regulating its intracellular localization (c-Myc—c-Myc-nick), modulating the activity of its antagonist protein (FOXO1/3), acetylating/deacetylating conserved sites, altering its protein-protein interactions, or combining all of these approaches? In our opinion, the development of in silico methods for predicting changes in the structure of proteins after their PTM and, accordingly, interaction with partner proteins, makes it possible to hope that these questions will be resolved. It is necessary to identify the mechanism by which the activity of the same proteins is regulated in different directions, which is necessary to increase neuronal survival at the early stage of acute injury or neurodegenerative process, to reduce neuronal death, and to increase their ability to regenerate at a later stage.
